# Supplementary material for: Splice-Junction-Based Mapping of Alternative Isoforms in the Human Proteome
Source: Cell Rep. Author manuscript; Available in PMC 2020 Jan 15. (PMC6961840; doi:10.1016/j.celrep.2019.11.026)
Supplement: 3 [file NIHMS1546469-supplement-3.zip › DF2/PXD0010154/Esophagus-30-Q9UEW8-VKEENPEGPPNANEDYR.pdf]

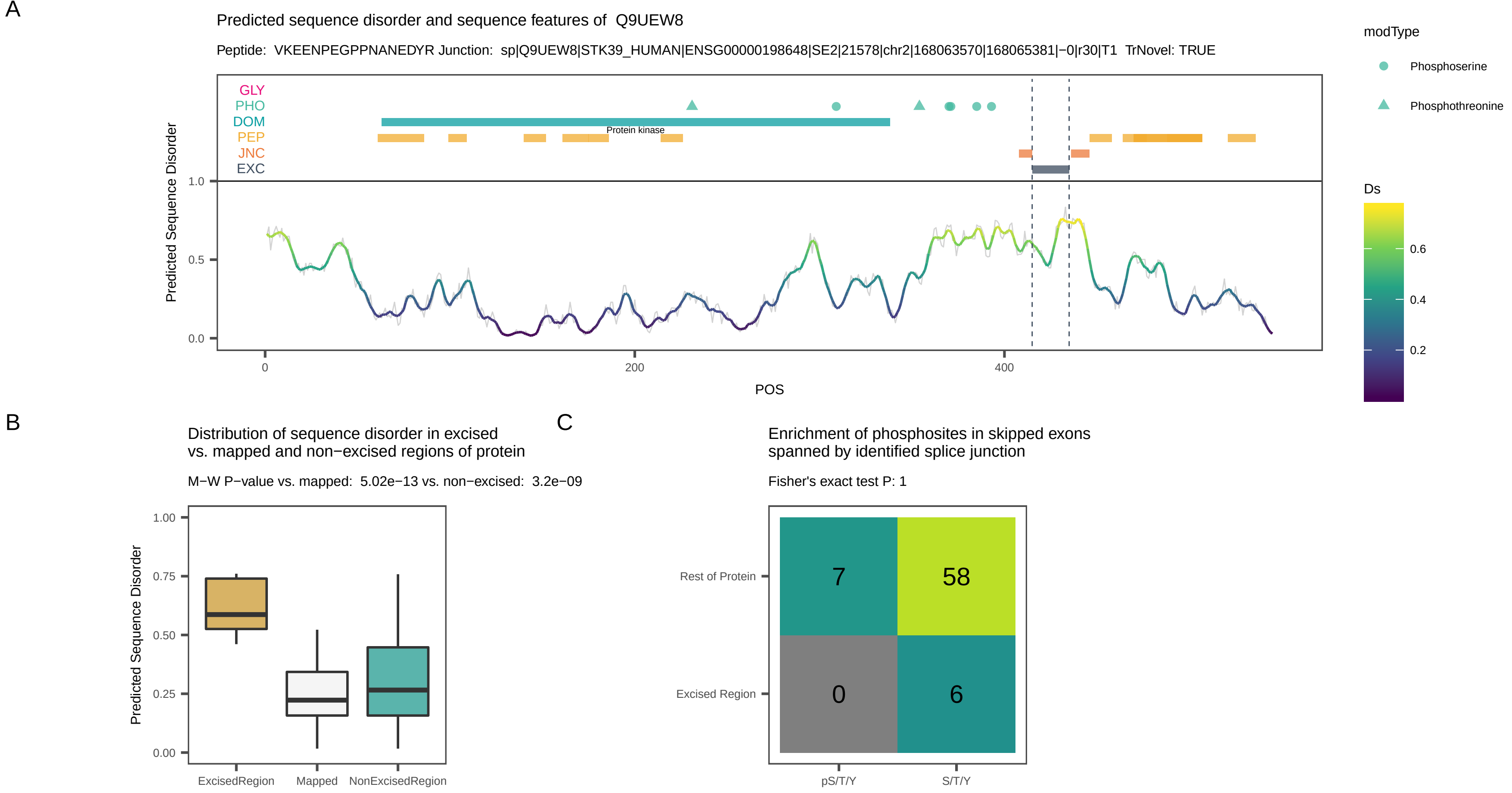
A
Predicted sequence disorder and sequence features of Q9UEW8
Peptide: VKEENPEGPPNANEDYR Junction: sp|Q9UEW8|STK39_HUMAN|ENSG00000198648|SE2|21578|chr2|168063570|168065381|−0|r30|T1 TrNovel: TRUE
modType
Phosphoserine
Phosphothreonine
GLY
PHO
DOM
PEP
JNC
EXC
Protein kinase
Predicted Sequence Disorder
1.0
0.5
0.0
0
200
400
POS
Ds
0.6
0.4
0.2
B
Distribution of sequence disorder in excised vs. mapped and non−excised regions of protein
M−W P−value vs. mapped: 5.02e−13 vs. non−excised: 3.2e−09
Predicted Sequence Disorder
1.00
0.75
0.50
0.25
0.00
ExcisedRegion
Mapped
NonExcisedRegion
C
Enrichment of phosphosites in skipped exons spanned by identified splice junction
Fisher's exact test P: 1
Rest of Protein
Excised Region
7
58
0
6
pS/T/Y
S/T/Y
